# Non-linear association between interpregnancy interval after vaginal delivery and singleton preterm birth: a retrospective cohort study

**DOI:** 10.1186/s12884-025-07373-x

**Published:** 2025-03-11

**Authors:** Tingting Zhuang, Yu Zhang, Xueli Ren, Qixin Pan, Jingli Sun

**Affiliations:** 1 Postgraduate Training Base of Jinzhou Medical University (General Hospital of Northern Theater Command), No.83, Wenhua Road, Shenhe District, Shenyang, 110016 China; 2 Department of Obstetrics and Gynecology, General Hospital of Northern Theater Command, No.83, Wenhua Road, Shenhe District, Shenyang, 110016 China; 3 Postgraduate Training Base of China Medical University (General Hospital of Northern Theater Command), No.83, Wenhua Road, Shenhe District, Shenyang, 110016 China; 4 Postgraduate Training Base of Dalian Medical University (General Hospital of Northern Theater Command), No.83, Wenhua Road, Shenhe District, Shenyang, 110016 China

**Keywords:** Interpregnancy interval, Preterm birth, Vaginal delivery, The National Vital Statistics System, Threshold effects

## Abstract

**Background:**

The association between interpregnancy interval (IPI) after vaginal delivery and preterm birth (PTB) in singleton has not been elucidated. The aim of this study is to investigate the association between interpregnancy interval after vaginal delivery and preterm birth.

**Methods:**

Birth data from the 2022 National Vital Statistics System (NVSS) were selected, and multinomial logistic regression models were used to determine the odds ratios (OR) and 95% confidence intervals (95% CI) for the association between IPI after vaginal delivery and PTB. A restricted cubic spline (RCS) model with multivariate adjustment was constructed with a 4-node OR curve to check for possible non-linear relationships. Threshold effect analysis was conducted using two-piecewise linear regression and a likelihood ratio test.

**Results:**

The study included a total of 1,517,106 subjects, with an average age of 30.56 ± 5.29 years. 113,613 subjects had PTB, while 1,403,493 did not. Compared to the reference group (18–23 months), IPI of ≤ 11 months and ≥ 24 months were associated with an increased risk of PTB. The RCS curve observed a J-shaped association between the IPI after vaginal delivery and PTB (P < 0.001), with the lowest point of PTB risk occurring at approximately 23 months. The effect values for < 23 months and ≥ 23 months were 0.975 (95% CI: 0.974 ~ 0.977, P < 0.001) and 1.006 (95% CI: 1.005 ~ 1.006, P < 0.001), respectively. The results of sensitivity analyses remained stable.

**Conclusion:**

In patients with a history of vaginal delivery, a J-shaped non-linear relationship was found between the IPI and the risk of PTB. IPIs of ≤ 11 months and ≥ 24 months were associated with an increased risk of PTB.

**Supplementary Information:**

The online version contains supplementary material available at 10.1186/s12884-025-07373-x.

## Background

The term "preterm birth" (PTB) is used to describe the onset of labor prior to the completion of 37 weeks of gestation [1]. In recent years, the prevalence of PTB has remained high on a global scale. It is estimated that the global incidence of PTB was approximately 13.4 million in 2020, representing approximately 9.9% of the total number of births [2]. It is important to note that there is a higher risk of short- or long-term complications, such as neurodevelopmental disorders, for preterm infants than for those born at full term. Furthermore, mortality and morbidity rates for preterm infants are negatively correlated with gestational age, with the highest risk being observed in those born at less than 28 weeks of gestation [3–5]. It has been demonstrated that preterm birth and its associated complications represent the primary cause of mortality among children under the age of five [6]. The occurrence of a PTB has a significant impact on the family unit, the economy, and the survival of subsequent preterm infants. The current range of interventions for PTB includes cervical cerclage, vaginal progesterone, and uterine supports [7], Nevertheless, the number of efficacious prophylactic strategies for PTB remains limited.

The interpregnancy interval (IPI) is the interval between the birth and the next pregnancy [8]. A number of studies have demonstrated that mothers with shorter IPI following live births exhibit a markedly elevated risk of PTB, low birth weight infants, babies of small for gestational age (SGA), birth defects, and perinatal mortality [9–13]. A recent study has indicated that there may be an association between short IPI and early infant neurodevelopmental delays [14]. Women with longer IPI have a significantly higher risk of non-transfused severe maternal death, PTB, gestational diabetes, pre-eclampsia, preterm rupture of membranes, and adverse perinatal outcomes [9, 15, 16]. IPI is a potential modifiable risk factor for PTB. The World Health Organization (WHO) has issued a recommendation that there should be an interval of at least 24 months between two full pregnancies in order to reduce the risk of adverse effects on the mother, the newborn, and the infant. Furthermore, it is advised that the duration of the interval between pregnancies be modified in accordance with the preceding birth [8]. Differences in mode of first birth may affect subsequent pregnancies [17, 18]. The repeated dilation and squeezing of the cervix during vaginal delivery may lead to cervical insufficiency and other changes in pelvic floor structures [19, 20], increasing the risk of PTB in subsequent pregnancies. Vaginal delivery is currently the most common mode of delivery. According to statistics, over 80% of babies worldwide are born in this way by 2023 [21]. Given this large population base, postpartum health and fertility guidance undoubtedly deserves our attention. Although there are some studies on the impact of delivery mode and IPI on reproductive health, research on the association between IPI and PTB in the population after vaginal delivery is limited. Therefore, this study conducted a large-scale retrospective cohort study to explore the association between different IPIs and PTB in the population after vaginal delivery, analyzing the optimal IPI with a lower risk of preterm birth and providing reference for the prevention of PTB and clinical practice in the population after vaginal delivery.

## Materials and methods

### Database

The data utilized in this retrospective cohort study were procured from the National Vital Statistics System (NVSS) 2022, a data repository established by the National Center for Health Statistics (NCHS), a division of the Centers for Disease Control and Prevention (CDC) (https://www.cdc.gov/nchs/nvss/about_nvss.htm), and are accessible to the public. The NVSS compiles data on births from birth certificates for all 50 states, 2 cities, and 5 territories in accordance with federal legislation. Since this study used publicly available data without any personally identifiable information, ethical approval was not necessary. In accordance with the stipulations set forth in the Vital Statistics Data User Agreement, this study has duly observed the conditions governing the utilization of the aforementioned data. The methodology and reporting of this study were conducted in accordance with the guidelines set forth in the Strengthening the Reporting of Observational Studies in Epidemiology (STROBE) statements [22].

### Study population

**Fig. 1:**
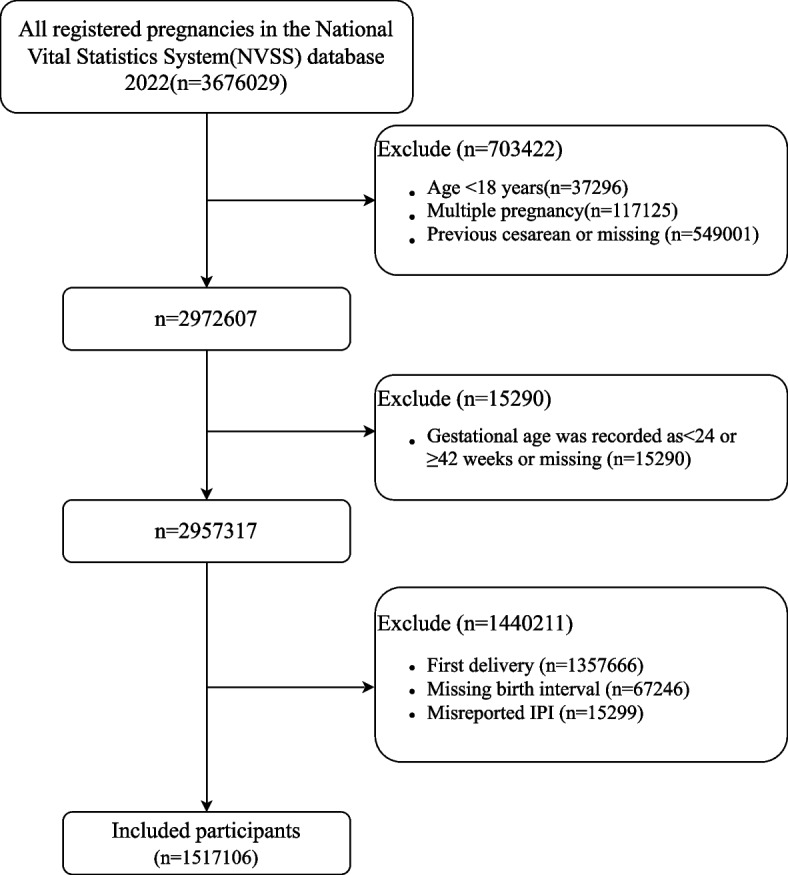
Flow chart of participant selection. Abbreviations: IPI, interpregnancy interval; NVSS, the National Vital Statistics System

The study population consisted of all women who gave birth in 2022. Women under the age of 18, multiple pregnancies, women with a history of previous cesarean section, gestational age < 24 weeks or ≥ 42 weeks, first pregnancy, unknown birth interval, and incorrect IPI were excluded from this study. The screening process is illustrated in Fig. 1.

### Exposure variable

The independent variable was IPI. The IPI is the interval between the birth and the next pregnancy [8]. In this instance, the gestational age was subtracted from the delivery date to calculate the date of conception. The IPI was categorized into 7 groups (< 6, 6–11, 12–17, 18–23, 24–35, 36–59, and ≥ 60 months) based on the findings of previous studies [11, 13, 23], with 18–23 months designated as the reference group.

### Covariates

The following data were collected from the NVSS: age (year), age at the prior birth (year), race (White, Black, Asian, or Other), marital status (married or unmarried), education level (less than an associate's degree or associate's degree and above), initiation of prenatal care (1st–3rd month, 4th–6th month, 7th–final month, or no prenatal care), parity before the current pregnancy (1, 2, or ≥ 3), intervening event during IPI (spontaneous or induced losses or ectopic pregnancy, (yes or no)), pre-pregnancy body mass index(BMI)(underweight(< 18.5 kg/m^2^), normal weight (18.5–24.9 kg/m^2^), overweight (25.0–29.9 kg/m^2^), or obesity (≥ 30 kg/m^2^)), weight gain during pregnancy (insufficient, adequate, or excessive), smoking before or during pregnancy (yes or no), pre-pregnancy or gestational diabetes (yes or no), pre-pregnancy or gestational hypertension (yes or no), hypertensive eclampsia (yes or no), previous history of preterm birth (yes or no), assisted reproductive technology (ART) (yes or no), infection during pregnancy (yes or no), chorioamnionitis (yes or no), mode of delivery (vaginal or C-section) and sex of infant (male or female). Classification of weight gain in pregnancy is based on the 2009 Institute of Medicine (IOM) guidelines [24].

The time at which covariates were determined was particularly important for individuals, as they may change over time and could be influenced by IPI [25, 26]. In order to better identify potential confounding factors, we used descriptive statistics and clinical experience to evaluate possible confounding factors guided by a directed acyclic graph (DAG) (Supplementary Fig. 1). The final model considered the following covariates: age at the prior birth, race, marital status, education level, intervening event during IPI, parity before the current pregnancy, pre-pregnancy BMI, smoking before pregnancy, pre-pregnancy diabetes, pre-pregnancy hypertension, and previous history of preterm birth.

### Outcomes

WHO defines PTB as the onset of labor prior to the completion of 37 weeks (< 259 days) of gestation. The term "PTB" can be further subdivided according to gestational age into the following categories: extremely preterm (< 28 weeks), very preterm (28–31 weeks), and moderate to late preterm (32–36 weeks) [1]. Information collected on gestational age is based on obstetric estimates (OE). During the initial prenatal visit, the clinician employs a process of estimation to determine the best obstetric date of delivery (BO-EDD). This estimation is based on the integration of all clinically relevant data, including information obtained from ultrasound imaging, the patient's last menstrual period (LMP), and a comprehensive physical examination [27]. The American College of Obstetricians and Gynecologists (ACOG) recommends that the optimal date of delivery should be determined as follows: (1) within 7 days of the date of the last menstrual period and the date assessed by ultrasound, on the basis of the LMP; (2) if the LMP is not known or the difference from the date estimated by ultrasound is 7 days, on the basis of the ultrasound; (3) if conception was conceived through assisted reproduction, on the basis of the date of conception [28]. Once the BO-EDD has been confirmed, it is used by the clinician to estimate the week of gestation and the gestational age at delivery. This obstetric-based estimate of PTB has been shown to have excellent specificity, positive predictive value, and negative predictive value in PTB [27].

### Statistical analysis

The statistical data were expressed as the mean value plus or minus the standard deviation (SD) for variables with a continuous distribution, while frequency counts and their corresponding percentages were used for categorical variables. In order to analyze categorical variables, the chi-square test was employed, whereas the one-way analysis of variance (ANOVA) was used to compare normally distributed continuous variables. Participant characteristics were described according to IPI groupings, and missing categories for covariates were added where necessary.

To investigate the association between IPI after vaginal delivery and PTB, this study conducted a multinomial logistic regression analysis. The multivariable model adjusted for covariates, including age at the prior birth, race, marital status, education level, intervening event during IPI, parity before the current pregnancy, pre-pregnancy BMI, smoking before pregnancy, pre-pregnancy diabetes, pre-pregnancy hypertension, and previous history of preterm birth. For the missing covariates, we used multiple imputation based on 5 iterations and the chained equation method in the R mice program to maximize statistical power and minimize potential bias due to missing data. The imputation model included all covariates without missing values and the IPI as the predictor variable.

In addition, we used a restricted cubic spline (RCS) model to construct a smooth curve to examine the potential non-linear dose–response relationship between IPI and PTB. In this model, participants with IPI within the range of 1–120 months were included, and all covariates were adjusted for. IPI was used as a continuous variable with four knots (5th, 35th, 65th, and 95th). Non-linearity was tested by including a quadratic term in the regression models.

If a non-linear association was observed, a two-piecewise linear regression model was used to find out the threshold effect, with adjustment for potential confounders. Likelihood ratio tests and bootstrap resampling methods were employed to determine the inflection point.

Subgroup analyses were conducted based on maternal age at the prior birth (< 35 years and ≥ 35 years), pre-pregnancy BMI, parity before the current pregnancy, and mode of delivery, with all covariates adjusted within these subgroups.

The intervening event during the IPI may lead to changes in the interval time and misclassification [26, 29–31]. In order to examine the robustness of the results, a series of sensitivity analyses were conducted in this study. Potential confounding factors (previous history of preterm birth, pre-pregnancy diabetes, pre-pregnancy hypertension), intervening event during the IPI, and SGA or large for gestational age (LGA) were excluded from the main analysis, and then the model was rerun to evaluate the impact of these complex pregnancy conditions on the association between IPI and PTB. Finally, sensitivity analyses were performed by excluding mothers with incomplete covariate data in order to examine the impact of missing covariate data on the analysis.

All statistical tests were two-sided, and differences were considered statistically significant at P < 0.05. All data were statistically analyzed using R 4.2.1 (http://www.R-project.org; The R Foundation, Vienna, Austria) and Free Statistics version 1.9.2.

## Results

### Baseline characteristics of study subjects

The total number of women who gave birth in the NVSS 2022 data was 367,6029. Based on the criteria used to include and exclude participants, 1,517,106 participants were ultimately enrolled. The flowchart illustrating the process of participant selection is presented in Fig. 1. Table 1 presented the baseline characteristics of the study population, grouped according to the IPI. Among these participants, there were 77,271 women (5.09%) in the < 6 months group, 181,759 women (11.98%) in the 6–11 months group, 217,008 women (14.30%) in the 12–17 months group, 182,289 women (12.02%) in the 18–23 months group, 252,535 women (16.65%) in the 24–35 months group, 277,615 women (18.30%) in the 36–59 months group, and 328,629 women (21.66%) in the ≥ 60 months group. The average age of these women was 30.56 ± 5.29 years, showing a gradual increase with longer IPIs. The majority were White (74.77%). There were 113,613 women (7.49%) who experienced preterm birth. Compared to the 18–23 months group, the incidence of PTB was significantly higher in the < 6 months group and the ≥ 60 months group (10.52% and 9.86%, respectively), displaying an overall J-shaped distribution across the different IPI groups.

**Table 1:** Population characteristics by categories of interpregnancy interval

Characteristic	IPI, month									*P*-value
	Total (*n* = 1,517,106)	< 6 months (*n* = 77,271)	6–11 months (*n* = 181,759)	12–17 months (*n* = 217,008)	18–23 months (*n* = 182,289)	24–35 months (*n* = 252,535)	36–59 months (*n* = 277,615)	≥ 60 months (*n* = 328,629)		
Age(year), Mean ± SD	30.56 ± 5.29	27.39 ± 5.28	28.83 ± 5.22	29.81 ± 5.11	30.18 ± 5.11	30.44 ± 5.14	30.72 ± 5.08		32.93 ± 4.83	< 0.001
Age at the prior birth(year), Mean ± SD	25.91 ± 5.26	25.71 ± 5.27	26.83 ± 5.22	27.39 ± 5.12	27.18 ± 5.11	26.78 ± 5.16	25.63 ± 5.11		23.32 ± 4.65	< 0.001
Race, n (%)										< 0.001
White	1,134,296 (74.77)	54,612 (70.68)	139,160 (76.56)	172,256 (79.38)	143,440 (78.69)	193,362 (76.57)	202,154 (72.82)		229,312 (69.78)	
Black	232,699 (15.34)	15,455 (20)	26,270 (14.45)	25,017 (11.53)	21,541 (11.82)	33,119 (13.11)	44,737 (16.11)		66,560 (20.25)	
Asian	85,006 (5.60)	2608 (3.38)	7934 (4.37)	11,194 (5.16)	10,268 (5.63)	15,915 (6.3)	18,655 (6.72)		18,432 (5.61)	
Other	65,105 (4.29)	4596 (5.95)	8395 (4.62)	8541 (3.94)	7040 (3.86)	10,139 (4.01)	12,069 (4.35)		14,325 (4.36)	
Marital status, n (%)										< 0.001
Married	842,937 (55.56)	33,845 (43.8)	106,416 (58.55)	141,192 (65.06)	117,629 (64.53)	154,655 (61.24)	148,412 (53.46)		140,788 (42.84)	
Unmarried	499,011 (32.89)	36,025 (46.62)	57,191 (31.47)	53,232 (24.53)	44,943 (24.65)	69,437 (27.5)	95,146 (34.27)		143,037 (43.53)	
Missing	175,158 (11.55)	7401 (9.58)	18,152 (9.99)	22,584 (10.41)	19,717 (10.82)	28,443 (11.26)	34,057 (12.27)		44,804 (13.63)	
Education level, n (%)										< 0.001
Less than an associate's degree	885,742 (58.38)	56,695 (73.37)	104,385 (57.43)	106,730 (49.18)	88,833 (48.73)	132,115 (52.32)	169,862 (61.19)		227,122 (69.11)	
Associate's degree and above	607,333 (40.03)	19,474 (25.2)	74,881 (41.2)	107,388 (49.49)	91,006 (49.92)	116,698 (46.21)	102,998 (37.1)		94,888 (28.87)	
Missing	24,031 (1.58)	1102 (1.43)	2493 (1.37)	2890 (1.33)	2450 (1.34)	3722 (1.47)	4755 (1.71)		6619 (2.01)	
Month prenatal care began, n (%)										< 0.001
1th to 3th month	1,138,547 (75.05)	48,315 (62.53)	131,126 (72.14)	166,263 (76.62)	142,442 (78.14)	196,446 (77.79)	210,682 (75.89)		243,273 (74.03)	
4th to 6th month	253,166 (16.69)	18,362 (23.76)	33,786 (18.59)	34,159 (15.74)	26,795 (14.7)	37,653 (14.91)	44,756 (16.12)		57,655 (17.54)	
7th to final month	67,295 (4.44)	5680 (7.35)	9056 (4.98)	8965 (4.13)	6890 (3.78)	9760 (3.86)	11,812 (4.25)		15,132 (4.6)	
No prenatal care	32,087 (2.12)	3195 (4.13)	4538 (2.5)	4063 (1.87)	3255 (1.79)	4578 (1.81)	5508 (1.98)		6950 (2.11)	
Missing	26,011 (1.71)	1719 (2.22)	3253 (1.79)	3558 (1.64)	2907 (1.59)	4098 (1.62)	4857 (1.75)		5619 (1.71)	
Parity before the current pregnancy, n (%)										< 0.001
1	35,270 (45.64)	93,420 (51.4)	118,585 (54.65)	100,493 (55.13)	135,972 (53.84)	140,012 (50.43)	163,951 (49.89)		35,270 (45.64)	
2	19,858 (25.7)	44,699 (24.59)	50,955 (23.48)	44,619 (24.48)	65,776 (26.05)	79,924 (28.79)	99,734 (30.35)		19,858 (25.7)	
≥ 3	21,970 (28.43)	43,248 (23.79)	47,003 (21.66)	36,764 (20.17)	50,229 (19.89)	57,126 (20.58)	64,284 (19.56)		21,970 (28.43)	
Missing	173 (0.22)	392 (0.22)	465 (0.21)	413 (0.23)	558 (0.22)	553 (0.2)	660 (0.2)		173 (0.22)	
Intervening event during IPI, n (%)										< 0.001
No	1,200,777 (79.15)	69,458 (89.89)	160,237 (88.16)	185,363 (85.42)	149,067 (81.78)	197,787 (78.32)	208,852 (75.23)		230,013 (69.99)	
Yes	176,224 (11.62)	838 (1.08)	6374 (3.51)	14,622 (6.74)	18,565 (10.18)	33,158 (13.13)	42,237 (15.21)		60,430 (18.39)	
Missing	140,105 (9.24)	6975 (9.03)	15,148 (8.33)	17,023 (7.84)	14,657 (8.04)	21,590 (8.55)	26,526 (9.55)		38,186 (11.62)	
BMI, n (%)										< 0.001
Underweight	36,653 (2.42)	1695 (2.19)	4917 (2.71)	6297 (2.9)	5046 (2.77)	6613 (2.62)	6326 (2.28)		5759 (1.75)	
Normal weight	562,507 (37.08)	24,665 (31.92)	71,351 (39.26)	93,731 (43.19)	77,538 (42.54)	100,241 (39.69)	97,267 (35.04)		97,714 (29.73)	
Overweight	423,663 (27.93)	21,545 (27.88)	49,672 (27.33)	57,084 (26.31)	48,669 (26.7)	69,417 (27.49)	78,898 (28.42)		98,378 (29.94)	
Obesity	464,733 (30.63)	27,457 (35.53)	52,121 (28.68)	55,859 (25.74)	47,850 (26.25)	71,625 (28.36)	89,744 (32.33)		120,077 (36.54)	
Missing	29,550 (1.95)	1909 (2.47)	3698 (2.03)	4037 (1.86)	3186 (1.75)	4639 (1.84)	5380 (1.94)		6701 (2.04)	
Weight gain during pregnancy, n (%)										< 0.001
Insufficient	416,757 (27.47)	24,406 (31.58)	51,779 (28.49)	59,032 (27.2)	48,846 (26.8)	68,169 (26.99)	77,095 (27.77)		87,430 (26.6)	
adequate	437,227 (28.82)	21,513 (27.84)	53,937 (29.68)	65,253 (30.07)	54,723 (30.02)	74,039 (29.32)	78,320 (28.21)		89,442 (27.22)	
Excessive	618,186 (40.75)	28,668 (37.1)	70,505 (38.79)	86,346 (39.79)	73,585 (40.37)	103,137 (40.84)	114,014 (41.07)		141,931 (43.19)	
Missing	44,936 (2.96)	2684 (3.47)	5538 (3.05)	6377 (2.94)	5135 (2.82)	7190 (2.85)	8186 (2.95)		9826 (2.99)	
Smoking before pregnancy, n (%)										< 0.001
No	1,433,061 (94.46)	71,275 (92.24)	172,676 (95)	208,512 (96.08)	174,898 (95.95)	241,190 (95.51)	261,567 (94.22)		302,943 (92.18)	
Yes	78,514 (5.18)	5638 (7.3)	8387 (4.61)	7774 (3.58)	6808 (3.73)	10,547 (4.18)	15,028 (5.41)		24,332 (7.4)	
Missing	5531 (0.36)	358 (0.46)	696 (0.38)	722 (0.33)	583 (0.32)	798 (0.32)	1020 (0.37)		1354 (0.41)	
Smoking during pregnancy, n (%)										< 0.001
No	1,457,151 (96.05)	72,488 (93.81)	174,853 (96.2)	210,609 (97.05)	177,015 (97.11)	244,581 (96.85)	266,553 (96.02)		311,052 (94.65)	
Yes	54,505 (3.59)	4425 (5.73)	6221 (3.42)	5682 (2.62)	4702 (2.58)	7176 (2.84)	10,057 (3.62)		16,242 (4.94)	
Missing	5450 (0.36)	358 (0.46)	685 (0.38)	717 (0.33)	572 (0.31)	778 (0.31)	1005 (0.36)		1335 (0.41)	
Pre-pregnancy diabetes, n (%)										< 0.001
No	1,502,434 (99.03)	76,656 (99.2)	180,528 (99.32)	215,549 (99.33)	180,998 (99.29)	250,475 (99.18)	274,766 (98.97)		323,462 (98.43)	
Yes	14,672 (0.97)	615 (0.8)	1231 (0.68)	1459 (0.67)	1291 (0.71)	2060 (0.82)	2849 (1.03)		5167 (1.57)	
Gestational diabetes, n (%)										< 0.001
No	1,394,702 (91.93)	72,142 (93.36)	170,322 (93.71)	202,708 (93.41)	170,088 (93.31)	233,700 (92.54)	253,746 (91.4)		291,996 (88.85)	
Yes	122,404 (8.07)	5129 (6.64)	11,437 (6.29)	14,300 (6.59)	12,201 (6.69)	18,835 (7.46)	23,869 (8.6)		36,633 (11.15)	
Pre-pregnancy hypertension, n (%)										< 0.001
No	1,477,353 (97.38)	75,286 (97.43)	177,912 (97.88)	212,840 (98.08)	178,646 (98)	246,697 (97.69)	270,341 (97.38)		315,631 (96.04)	
Yes	39,753 (2.62)	1985 (2.57)	3847 (2.12)	4168 (1.92)	3643 (2)	5838 (2.31)	7274 (2.62)		12,998 (3.96)	
Gestational hypertension, n (%)										< 0.001
No	1,407,179 (92.75)	72,330 (93.61)	170,434 (93.77)	203,468 (93.76)	170,378 (93.47)	235,367 (93.2)	256,741 (92.48)		298,461 (90.82)	
Yes	109,927 (7.25)	4941 (6.39)	11,325 (6.23)	13,540 (6.24)	11,911 (6.53)	17,168 (6.8)	20,874 (7.52)		30,168 (9.18)	
Hypertension eclampsia, n (%)										< 0.001
No	1,514,147 (99.80)	77,146 (99.84)	181,462 (99.84)	216,692 (99.85)	181,987 (99.83)	252,115 (99.83)	277,045 (99.79)		327,700 (99.72)	
Yes	2959 (0.20)	125 (0.16)	297 (0.16)	316 (0.15)	302 (0.17)	420 (0.17)	570 (0.21)		929 (0.28)	
Previous preterm birth, n (%)										< 0.001
No	1,437,954 (94.78)	71,727 (92.83)	171,893 (94.57)	206,456 (95.14)	173,559 (95.21)	239,811 (94.96)	263,139 (94.79)		311,369 (94.75)	
Yes	79,152 (5.22)	5544 (7.17)	9866 (5.43)	10,552 (4.86)	8730 (4.79)	12,724 (5.04)	14,476 (5.21)		17,260 (5.25)	
ART, n (%)										< 0.001
No	1,502,372 (99.03)	77,139 (99.83)	180,711 (99.42)	214,707 (98.94)	180,167 (98.84)	249,596 (98.84)	274,729 (98.96)		325,323 (98.99)	
Yes	14,162 (0.93)	121 (0.16)	999 (0.55)	2222 (1.02)	2033 (1.12)	2820 (1.12)	2765 (1)		3202 (0.97)	
Missing	572 (0.04)	11 (0.01)	49 (0.03)	79 (0.04)	89 (0.05)	119 (0.05)	121 (0.04)		104 (0.03)	
Infections, n (%)										< 0.001
No	1,472,873 (97.08)	74,165 (95.98)	176,585 (97.15)	211,934 (97.66)	177,929 (97.61)	245,885 (97.37)	269,097 (96.93)		317,278 (96.55)	
Yes	38,841 (2.56)	2751 (3.56)	4477 (2.46)	4266 (1.97)	3739 (2.05)	5817 (2.3)	7599 (2.74)		10,192 (3.1)	
Missing	5392 (0.36)	355 (0.46)	697 (0.38)	808 (0.37)	621 (0.34)	833 (0.33)	919 (0.33)		1159 (0.35)	
Chorioamnionitis, n (%)										< 0.001
No	1,508,088 (99.41)	76,927 (99.55)	180,985 (99.57)	216,132 (99.6)	181,472 (99.55)	251,255 (99.49)	275,796 (99.34)		325,521 (99.05)	
Yes	8274 (0.55)	300 (0.39)	682 (0.38)	767 (0.35)	707 (0.39)	1176 (0.47)	1689 (0.61)		2953 (0.9)	
Missing	744 (0.05)	44 (0.06)	92 (0.05)	109 (0.05)	110 (0.06)	104 (0.04)	130 (0.05)		155 (0.05)	
Mode of delivery, n (%)										< 0.001
Vaginal	1,328,095 (87.54)	69,603 (90.08)	164,537 (90.52)	196,395 (90.5)	163,821 (89.87)	224,050 (88.72)	241,481 (86.98)		268,208 (81.61)	
C-Section	188,267 (12.41)	7624 (9.87)	17,121 (9.42)	20,468 (9.43)	18,369 (10.08)	28,366 (11.23)	36,025 (12.98)		60,294 (18.35)	
Missing	744 (0.05)	44 (0.06)	101 (0.06)	145 (0.07)	99 (0.05)	119 (0.05)	109 (0.04)		127 (0.04)	
Sex of infant, n (%)										0.485
Male	774,243 (51.03)	39,265 (50.81)	92,782 (51.05)	110,909 (51.11)	92,743 (50.88)	129,196 (51.16)	141,601 (51.01)		167,747 (51.04)	
Female	742,863 (48.97)	38,006 (49.19)	88,977 (48.95)	106,099 (48.89)	89,546 (49.12)	123,339 (48.84)	136,014 (48.99)		160,882 (48.96)	
Preterm birth, n (%)										< 0.001
No	1,403,493 (92.51)	69,143 (89.48)	169,066 (93.02)	204,134 (94.07)	171,736 (94.21)	236,053 (93.47)	257,124 (92.62)		296,237 (90.14)	
Yes	113,613 (7.49)	8128 (10.52)	12,693 (6.98)	12,874 (5.93)	10,553 (5.79)	16,482 (6.53)	20,491 (7.38)		32,392 (9.86)	
< 28 weeks	3634 (0.24)	277 (0.36)	379 (0.21)	351 (0.16)	260 (0.14)	464 (0.18)	636 (0.23)		1267 (0.39)	
28-31 weeks	8253 (0.54)	638 (0.83)	876 (0.48)	794 (0.37)	661 (0.36)	1074 (0.43)	1447 (0.52)		2763 (0.84)	
32-36 weeks	101,726 (6.71)	7213 (9.33)	11,438 (6.29)	11,729 (5.4)	9632 (5.28)	14,944 (5.92)	18,408 (6.63)		28,362 (8.63)	

*Abbreviations:* *IPI* Interpregnancy interval, *SD* Standard deviation, *BMI* Body mass index (calculated as weight in kilograms divided by the square of height in meters), *ART* Assisted reproductive technology

### Association between IPI and PTB

A multinomial logistic regression model was used to assess the association between IPI after vaginal delivery and PTB (Table 2). The results suggested, compared to the reference group, intervals of ≤ 11 months or ≥ 24 months were associated with an increased risk of varying categories of preterm birth, with a trend of increasing risk as the IPI shortened or lengthened ( Moderate to late preterm birth: < 6 months, 1.58 (1.53 ~ 1.63); 6–11 months, 1.14 (1.11 ~ 1.18); 24–35 months, 1.10 (1.07 ~ 1.13); 36–59 months, 1.19 (1.16 ~ 1.22); ≥ 60 months, 1.52 (1.48 ~ 1.56). Very preterm birth: < 6 months, 1.90 (1.70 ~ 2.13); 6–11 months, 1.25 (1.13 ~ 1.38); 24–35 months, 1.14 (1.04 ~ 1.26); 36–59 months, 1.33 (1.21 ~ 1.46); ≥ 60 months, 2.09 (1.91 ~ 2.28). Extremely preterm birth: < 6 months, 2.07 (1.74 ~ 2.45); 6–11 months, 1.37 (1.17 ~ 1.60); 24–35 months, 1.24 (1.07 ~ 1.45); 36–59 months, 1.45 (1.25 ~ 1.68); ≥ 60 months, 2.35 (2.05 ~ 2.69)).

**Table 2:** The logistic regression of interpregnancy interval associated with preterm birth

IPI groups	n. Total	n. Event %	Preterm birth, OR (95% CI)	Preterm birth categories		
				Moderate to late preterm birth, OR (95% CI)	Very preterm birth, OR (95% CI)	Extremely preterm birth, OR (95% CI)
< 6 months	77,271	8128 (10.5)	1.61 (1.56 ~ 1.66)	1.58 (1.53 ~ 1.63)	1.90 (1.70 ~ 2.13)	2.07 (1.74 ~ 2.45)
6–11 months	181,759	12,693 (7.0)	1.16 (1.12 ~ 1.19)	1.14 (1.11 ~ 1.18)	1.25 (1.13 ~ 1.38)	1.37 (1.17 ~ 1.60)
12–17 months	217,008	12,874 (5.9)	1.03 (1.00 ~ 1.05)	1.02 (1.00 ~ 1.05)	1.01 (0.91 ~ 1.12)	1.14 (0.97 ~ 1.34)
18–23 months	182,289	10,553 (5.8)	1 (Ref)	1 (Ref)	1 (Ref)	1 (Ref)
24–35 months	252,535	16,482 (6.5)	1.11 (1.08 ~ 1.13)	1.10 (1.07 ~ 1.13)	1.14 (1.04 ~ 1.26)	1.24 (1.07 ~ 1.45)
36–59 months	277,615	20,491 (7.4)	1.20 (1.17 ~ 1.23)	1.19 (1.16 ~ 1.22)	1.33 (1.21 ~ 1.46)	1.45 (1.25 ~ 1.68)
≥ 60 months	328,629	32,392 (9.9)	1.57 (1.54 ~ 1.61)	1.52 (1.48 ~ 1.56)	2.09 (1.91 ~ 2.28)	2.35 (2.05 ~ 2.69)

Adjusted for age at the prior birth, race, marital status, education level, intervening event during IPI, parity before the current pregnancy, pre-pregnancy BMI, smoking before pregnancy, pre-pregnancy diabetes, pre-pregnancy hypertension, and previous history of preterm birth

*Abbreviations*: *IPI* Interpregnancy interval, *CI* Confidence interval, *OR* Odds ratio, *Ref* Reference, *BMI* Body mass index

**Fig. 2:**
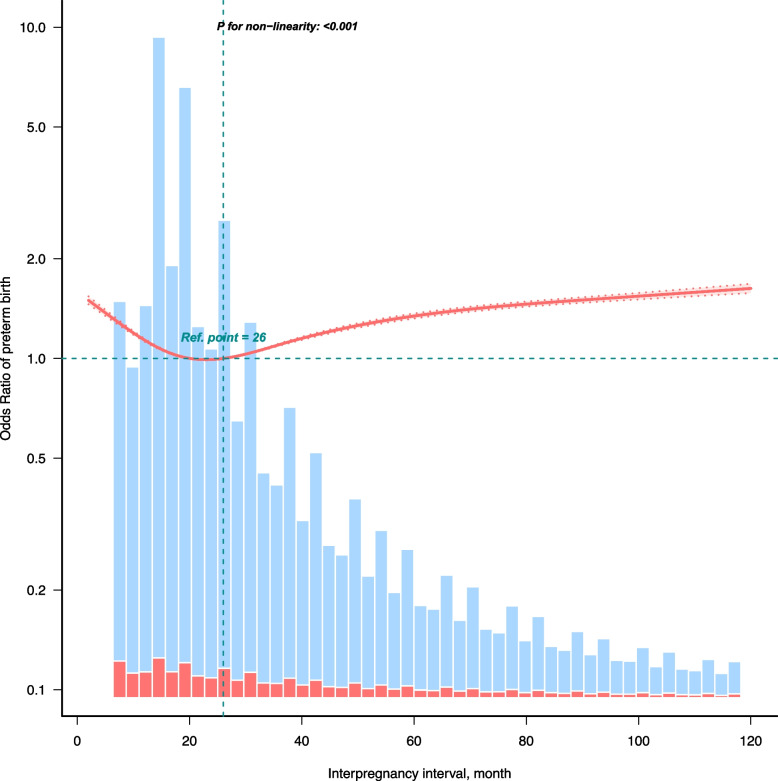
Smooth curve fitting of the relationship between interpregnancy interval and preterm birth. The solid line and dashed lines represent the predicted values and their corresponding 95% confidence intervals. Adjusted for age at the prior birth, race, marital status, education level, intervening event during IPI, parity before the current pregnancy, pre-pregnancy BMI, smoking before pregnancy, pre-pregnancy diabetes, pre-pregnancy hypertension, and previous history of preterm birth. Only data for the IPI in the range of 1–120 months are shown (*n* = 1,439,050). Abbreviations: IPI, interpregnancy interval; BMI, body mass index

Through multinomial logistic regression analysis and smooth curve fitting, the observed association between IPI and the risk of PTB was non-linear. The RCS analysis suggested a J-shaped association (non-linear *P* < 0.001, Fig. 2). After complete adjustment for covariates, a two-piecewise linear regression model indicated that the lowest risk point was approximately 23 months (Table 3). Among participants with intervals of < 23 months, each unit increase in IPI was associated with a 2.5% reduction in the risk of PTB (OR: 0.975, 95% CI: 0.974 ~ 0.977, *P* < 0.001). Conversely, among participants with intervals of ≥ 23 months, each unit increase was associated with a 0.6% increase in risk (OR: 1.006, 95% CI: 1.005 ~ 1.006, *P* < 0.001). These findings indicated that both short and long IPIs were associated with an increased risk of PTB, with an interval of approximately 23 months potentially associated with the lowest risk.

**Table 3:** Threshold effect analysis of the association between interpregnancy interval and preterm birth

	OR	95% CI	*P*-value
Turning point (month)	23		
Two-piecewise linear regression model			
< 23	0.975	(0.974 ~ 0.977)	< 0.001
≥ 23	1.006	(1.005 ~ 1.006)	< 0.001
Likelihood Ratio test			< 0.001

Adjusted for age at the prior birth, race, marital status, education level, intervening event during IPI, parity before the current pregnancy, pre-pregnancy BMI, smoking before pregnancy, pre-pregnancy diabetes, pre-pregnancy hypertension, and previous history of preterm birth

Only data for IPI in the range of 1–120 months are shown (*n* = 1,439,050)

*Abbreviations*: *IPI* Interpregnancy interval, *CI* Confidence interval, *OR* Odds ratio, *BMI* Body mass index

### Subgroup analysis and sensitivity analysis

Regarding the subgroup analysis by maternal age at the prior birth, pre-pregnancy BMI, parity before the current pregnancy, and mode of delivery (Supplementary Figs. 2, 3, 4, and 5). An increased risk of preterm birth at 32–36 weeks was observed in almost all subgroups when IPI was ≤ 11 months or ≥ 24 months, except the underweight subgroup, with slight differences between subgroups in the risk of preterm birth at < 32 weeks. Interestingly, no statistical significance was observed for the 6–11 months in the subgroup with a previous delivery age ≥ 35 years.

SGA and LGA were defined as neonates with birth weights less than the 10th percentile and equal to or more than the 90th percentile for the same gestational age, respectively [32–35]. The presence of SGA and LGA may be related to confounders that could not be included in our study [36–38]. Sensitivity analyses excluded participants with potential risk confounders (previous history of preterm birth, pre-pregnancy diabetes, pre-pregnancy hypertension), intervening event during the IPI, and delivery of SGA or LGA neonates (Table 4, Supplementary Fig. 6). Furthermore, although the frequency of missing data is low for almost all covariates, data on marital status is missing for 175,158 women (11.55%) (Table 1). Participants with missing covariates were also excluded from the sensitivity analyses, and the results were similar to the main analyses, with no evidence of a change in the J-shaped relationship between IPI and risk of PTB after vaginal delivery.

**Table 4:** Sensitivity analysis for the association of interpregnancy interval with preterm birth

Variable	n. Total	Moderate to late preterm birth		Very preterm birth		Extremely preterm birth	
		OR (95% CI)	*P*-value	OR (95% CI)	*P*-value	OR (95% CI)	*P*-value
**Without previous history of preterm birth (*n* = 1,437,954)**							
< 6 months	71,727	1.60 (1.54 ~ 1.66)	< 0.001	1.82 (1.60 ~ 2.06)	< 0.001	1.70 (1.40 ~ 2.08)	< 0.001
6–11 months	171,893	1.14 (1.11 ~ 1.18)	< 0.001	1.18 (1.05 ~ 1.32)	0.005	1.25 (1.04 ~ 1.49)	0.014
12–17 months	206,456	1.02 (0.99 ~ 1.05)	0.303	0.95 (0.85 ~ 1.07)	0.415	1.03 (0.86 ~ 1.23)	0.750
18–23 months	173,559	1(Ref)		1(Ref)		1(Ref)	
24–35 months	239,811	1.10 (1.07 ~ 1.13)	< 0.001	1.15 (1.04 ~ 1.29)	0.009	1.15 (0.97 ~ 1.36)	0.106
36–59 months	263,139	1.20 (1.17 ~ 1.24)	< 0.001	1.29 (1.16 ~ 1.43)	< 0.001	1.35 (1.15 ~ 1.58)	< 0.001
≥ 60 months	311,369	1.56 (1.52 ~ 1.60)	< 0.001	2.05 (1.86 ~ 2.25)	< 0.001	2.20 (1.89 ~ 2.55)	< 0.001
**Without pre-pregnancy diabetes (*n* = 1,502,434)**							
< 6 months	76,656	1.57 (1.52 ~ 1.62)	< 0.001	1.89 (1.69 ~ 2.11)	< 0.001	2.04 (1.72 ~ 2.42)	< 0.001
6–11 months	180,528	1.14 (1.11 ~ 1.17)	< 0.001	1.23 (1.11 ~ 1.37)	< 0.001	1.36 (1.16 ~ 1.59)	< 0.001
12–17 months	215,549	1.02 (1.00 ~ 1.05)	0.105	1.01 (0.91 ~ 1.12)	0.917	1.13 (0.96 ~ 1.33)	0.127
18–23 months	180,998	1(Ref)		1(Ref)		1(Ref)	
24–35 months	250,475	1.10 (1.07 ~ 1.13)	< 0.001	1.13 (1.03 ~ 1.25)	0.012	1.23 (1.05 ~ 1.43)	0.009
36–59 months	274,766	1.19 (1.16 ~ 1.22)	< 0.001	1.31 (1.20 ~ 1.44)	< 0.001	1.40 (1.21 ~ 1.62)	< 0.001
≥ 60 months	323,462	1.51 (1.47 ~ 1.55)	< 0.001	2.01 (1.85 ~ 2.20)	< 0.001	2.21 (1.93 ~ 2.54)	< 0.001
**Without pre-pregnancy hypertension (*n* = 1,477,353)**							
< 6 months	75,286	1.59 (1.54 ~ 1.64)	< 0.001	1.91 (1.70 ~ 2.13)	< 0.001	2.05 (1.72 ~ 2.45)	< 0.001
6–11 months	177,912	1.15 (1.11 ~ 1.18)	< 0.001	1.25 (1.13 ~ 1.39)	< 0.001	1.39 (1.18 ~ 1.63)	< 0.001
12–17 months	212,840	1.03 (1.00 ~ 1.06)	0.088	1.01 (0.91 ~ 1.12)	0.901	1.13 (0.96 ~ 1.33)	0.150
18–23 months	178,646	1(Ref)		1(Ref)		1(Ref)	
24–35 months	246,697	1.10 (1.07 ~ 1.13)	< 0.001	1.13 (1.02 ~ 1.24)	0.021	1.21 (1.03 ~ 1.41)	0.018
36–59 months	270,341	1.18 (1.15 ~ 1.21)	< 0.001	1.28 (1.17 ~ 1.41)	< 0.001	1.38 (1.18 ~ 1.60)	< 0.001
≥ 60 months	315,631	1.50 (1.46 ~ 1.53)	< 0.001	1.97 (1.80 ~ 2.16)	< 0.001	2.17 (1.89 ~ 2.50)	< 0.001
**Without intervening event during IPI (*n* = 1,321,347)**							
< 6 months	76,341	1.56 (1.51 ~ 1.62)	< 0.001	1.93 (1.72 ~ 2.16)	< 0.001	2.14 (1.79 ~ 2.56)	< 0.001
6–11 months	174,818	1.14 (1.10 ~ 1.17)	< 0.001	1.26 (1.13 ~ 1.40)	< 0.001	1.43 (1.21 ~ 1.70)	< 0.001
12–17 months	201,117	1.02 (0.99 ~ 1.05)	0.237	1.02 (0.91 ~ 1.14)	0.748	1.19 (1.00 ~ 1.42)	0.044
18–23 months	162,058	1(Ref)		1(Ref)		1(Ref)	
24–35 months	216,234	1.10 (1.07 ~ 1.13)	< 0.001	1.16 (1.04 ~ 1.28)	0.007	1.33 (1.12 ~ 1.57)	0.001
36–59 months	230,818	1.19 (1.15 ~ 1.22)	< 0.001	1.32 (1.19 ~ 1.46)	< 0.001	1.50 (1.27 ~ 1.76)	< 0.001
≥ 60 months	259,961	1.51 (1.47 ~ 1.55)	< 0.001	2.09 (1.90 ~ 2.29)	< 0.001	2.31 (1.99 ~ 2.69)	< 0.001
**Without previous preterm birth, pre-pregnancy diabetes, pre-pregnancy hypertension and intervening event during IPI (*n* = 1,217,716)**							
< 6 months	68,815	1.60 (1.54 ~ 1.66)	< 0.001	1.81 (1.59 ~ 2.07)	< 0.001	1.84 (1.49 ~ 2.28)	< 0.001
6–11 months	161,430	1.14 (1.11 ~ 1.18)	< 0.001	1.16 (1.02 ~ 1.31)	0.020	1.39 (1.15 ~ 1.69)	0.001
12–17 months	187,269	1.02 (0.98 ~ 1.05)	0.361	0.95 (0.84 ~ 1.08)	0.454	1.11 (0.91 ~ 1.36)	0.286
18–23 months	150,934	1(Ref)		1(Ref)		1(Ref)	
24–35 months	200,239	1.10 (1.07 ~ 1.14)	< 0.001	1.13 (1.01 ~ 1.27)	0.040	1.28 (1.06 ~ 1.55)	0.010
36–59 months	212,790	1.21 (1.17 ~ 1.25)	< 0.001	1.22 (1.08 ~ 1.36)	0.001	1.42 (1.19 ~ 1.71)	< 0.001
≥ 60 months	236,239	1.56 (1.51 ~ 1.60)	< 0.001	1.98 (1.78 ~ 2.21)	< 0.001	2.23 (1.88 ~ 2.65)	< 0.001
**Without SGA or LGA (*n* = 1,217,760)**							
< 6 months	63,033	1.58 (1.53 ~ 1.64)	< 0.001	1.99 (1.76 ~ 2.25)	< 0.001	1.98 (1.65 ~ 2.38)	< 0.001
6–11 months	146,425	1.15 (1.12 ~ 1.19)	< 0.001	1.32 (1.18 ~ 1.48)	< 0.001	1.33 (1.12 ~ 1.58)	0.001
12–17 months	174,314	1.02 (0.99 ~ 1.05)	0.198	1.06 (0.94 ~ 1.19)	0.349	1.12 (0.95 ~ 1.34)	0.181
18–23 months	146,172	1(Ref)		1(Ref)		1(Ref)	
24–35 months	202,101	1.09 (1.05 ~ 1.12)	< 0.001	1.14 (1.02 ~ 1.28)	0.017	1.24 (1.05 ~ 1.45)	0.011
36–59 months	222,529	1.17 (1.14 ~ 1.20)	< 0.001	1.32 (1.19 ~ 1.47)	< 0.001	1.35 (1.15 ~ 1.58)	< 0.001
≥ 60 months	263,186	1.49 (1.45 ~ 1.53)	< 0.001	2.03 (1.84 ~ 2.24)	< 0.001	2.15 (1.86 ~ 2.49)	< 0.001
**Without missing covariates (*n* = 1,182,573)**							
< 6 months	61,477	1.57 (1.52 ~ 1.63)	< 0.001	1.91 (1.68 ~ 2.18)	< 0.001	2.2 (1.81 ~ 2.68)	< 0.001
6–11 months	145,674	1.15 (1.11 ~ 1.19)	< 0.001	1.25 (1.11 ~ 1.41)	< 0.001	1.31 (1.09 ~ 1.58)	0.005
12–17 months	174,316	1.03 (1.00 ~ 1.06)	0.056	1.09 (0.97 ~ 1.23)	0.159	1.19 (0.99 ~ 1.43)	0.063
18–23 months	145,648	1(Ref)		1(Ref)		1(Ref)	
24–35 months	199,294	1.11 (1.07 ~ 1.14)	< 0.001	1.15 (1.03 ~ 1.29)	0.014	1.27 (1.06 ~ 1.51)	0.009
36–59 months	213,700	1.20 (1.16 ~ 1.23)	< 0.001	1.36 (1.22 ~ 1.51)	< 0.001	1.46 (1.24 ~ 1.73)	< 0.001
≥ 60 months	242,464	1.50 (1.46 ~ 1.54)	< 0.001	2.00 (1.81 ~ 2.22)	< 0.001	2.30 (1.96 ~ 2.70)	< 0.001

Age at the prior birth, race, marital status, education level, intervening event during IPI, parity before the current pregnancy, pre-pregnancy BMI, smoking before pregnancy, pre-pregnancy diabetes, pre-pregnancy hypertension, and previous history of preterm birth were adjusted for in the model except when the variable was excluded

*Abbreviations: CI* Confidence interval, *OR* Odds ratio, *Ref* Reference, *IPI* Interpregnancy interval, *BMI* Body mass index, *SGA* Small for gestational age, *LGA* Large for gestational age

## Discussion

In this large, nationally representative retrospective cohort study, the IPI following vaginal delivery was found to be an independent risk factor for PTB. Trends in effect values for different IPI groups were not equal, suggesting that there may be a non-linear relationship between IPI and PTB. Both ≤ 11 months and ≥ 24 months IPIs were associated with an increased risk of PTB. The RCS analysis revealed evidence of a J-shaped association between IPI after vaginal delivery and the risk of PTB. Threshold effect analysis found that an IPI of around 23 months may be associated with the lowest risk of PTB.

In the study conducted by Palmsten et al., a similar non-linear relationship between IPI and risk of PTB was found [39–41].The findings indicated that both short and long IPI were associated with a high risk of PTB, which is consistent with the results of our study. However, the IPIs with the minimal risk are not the same, and the discrepancy may be attributed to various factors, including differing study populations, demographic characteristics, and adjustment factors. To date, there are very few studies that have proceeded to investigate the threshold effect of IPI on the risk of PTB. Janša's study, which included 2,723 women, demonstrated that an IPI of 15 months from the last delivery was associated with a reduced risk of PTB. However, the analytic model was adjusted for only two factors, namely age and BMI [42]. This may have resulted in the potential for other confounding factors to influence the results being overlooked. In addition, no statistically significant difference between IPI and risk of PTB was observed in a Chinese population-based study after adjusting for confounders, which may be limited by the single-center, small-sample size limitation [43]. Ball, Hanley et al. [44, 45] employed a distinct study design from that of previous studies, utilizing the same maternal comparisons and concluding that the risk of PTB was unaffected by both shorter and longer IPI. The proponents of this approach maintain that it circumvents the necessity for adjustments based on factors such as genetic predisposition, lifestyle, or social conditions. However, the statistical analyses of the two studies in question were based on women whose three deliveries were all singleton live births. As a result, the findings may have been influenced by selection bias. There were differences in both second- and third-trimester pregnancy intervals and pregnancy outcomes, which were not controlled for by including variables such as the number of births, maternal age, and changes in BMI as independent effects. In this study, to validate these results and to assess whether the association between IPI after vaginal delivery and risk of PTB varied with maternal age at the prior birth, pre-pregnancy BMI, and parity before the current pregnancy, subgroup analyses were performed. The analyses showed that an increased risk of PTB at 32–36 weeks was observed in almost all subgroups when IPI was ≤ 11 months or ≥ 24 months, except the underweight subgroup. In addition, a series of sensitivity analyses were performed in this study to verify the robustness of the results.

Delivery patterns in previous pregnancies may be associated with subsequent pregnancy outcomes [17, 18]. In recent years, several cohort studies have examined the potential association between previous mode of delivery and the risk of PTB in subsequent pregnancies. Although a large number of studies have suggested that delivery by caesarean section in the first pregnancy is more likely to increase the risk of PTB in subsequent pregnancies compared with previous vaginal delivery [46, 47]. Interestingly, the study by Zhang et al. did not find that PTB at < 32 weeks of age was associated with a different prior mode of delivery [47]. Still, other cohort studies have noted that there is insufficient evidence to establish a clear link between first caesarean section and subsequent PTB [48]. Particularly after adjusting for a variety of confounders, some researchers have even observed that prior cesarean experience may actually reduce the risk of PTB in subsequent pregnancies [18]. Therefore, it would be interesting to increase analyses of the differentiation of PTB by cesarean or vaginal delivery. The ACOG suggests that pregnant women should be adequately assessed prenatally for their willingness and time plan for subsequent pregnancies and recommends an 18-month to 5-year interval between live births for re-pregnancy [49, 50]. However, there are limited recommendations for differentiation between different modes of delivery. A prospective study by Kristen H. et al. [17]with up to 36 months of follow-up in a population after different modes of delivery found that women with previous caesarean sections had lower rates of unprotected conception and live births in subsequent pregnancies compared with vaginal births, which may lead to a longer time between pregnancies. A recent study on the correlation between IPI and adverse maternal outcomes after cesarean delivery found no correlation between IPI and the risk of PTB [51]. This study focuses on the population after vaginal delivery to add to and inform the limited research in this area.

The evidence base regarding the impact of IPI on PTB is limited. On the one hand, some studies have indicated that the elevated risk of PTB among individuals with shorter IPI may be associated with socioeconomic status and unstable lifestyles [52]. Other studies have proposed that the deterioration of physiological functions resulting from nutrient depletion and deficiencies may contribute to an increased risk of PTB among individuals with shorter IPI [41, 53]. In a recent study, Costello and colleagues observed an association between incomplete restoration of the vaginal ecosystem and an elevated risk of PTB [54, 55]. Lactobacillus, especially L. crispatus, in the bacterial colonization of the vagina, which reduces vaginal pH, participates in the construction of the defense system and promotes a dynamic equilibrium and has been associated with a lower risk of delivering preterm infants, whereas a history of short-term pregnancies may have led to its loss of dominance, producing a localized pro-inflammatory cytokine response that can lead to adverse maternal and infant outcomes [54]. In addition, a meta-analysis of US population data showed that most pregnancies with a very short time between conception and the start of gestation are unplanned. Unplanned pregnancies have also been found to be associated with higher levels of maternal stress during pregnancy, which may raise the risk of an early birth [56]. On the other hand, it has been postulated that longer IPIs are related to an elevated risk of PTB. This is based on two main hypotheses. The first is the "physiological regression hypothesis," which suggests that the physiological adaptations of the previous pregnancy are lost, causing a return to an infertile state [10]. The second hypothesis posits that longer IPI may be attributed to infertility-related factors, thereby increasing the risk of PTB [57]. In addition, certain non-measurable factors, such as anatomical factors, metabolic factors, or other maternal disorders, may contribute to poor birth and pregnancy outcomes [10]. A recent Japanese population-based study by Tanigawa and colleagues concluded that inadequate folic acid intake during pregnancy may be associated with an increased incidence of PTB at both short and long IPI [58]. Adequate and reasonable folic acid supplementation during pregnancy may help reduce the risks associated with IPI.

This study is strengthened by its national scope and the size of the sample. Notwithstanding the constraints, it seems that optimizing the IPI following vaginal delivery may potentially diminish the likelihood of PTB. It is recommended that women be aware of the relationship between the duration of the interval between pregnancies after vaginal delivery and the risk of PTB in the next pregnancy, particularly in those with a history of preterm vaginal deliveries, where the probability of PTB in a subsequent pregnancy via vaginal delivery is relatively high. It is further encouraged that they adhere to the optimal interval. In addition, this study was based on clinically readily available baseline information, which may enhance the generalizability of the findings. Furthermore, it is important to acknowledge that this research has certain limitations. Firstly, the NVSS birth data lack sufficient detail to distinguish between spontaneous and medically induced PTB. Secondly, this study attempted to include as many pregnancy outcomes and potential influencing factors as possible, as documented in the medical records. However, due to a lack of pertinent data in the database, some confounding factors, including amniotic fluid status, history of cervical surgery, cervical ripeness, and information on vitamin, iron, and folic acid supplementation, could not be analyzed. To ensure the reliability of the results, a series of logistic regression analyses and subgroup analyses were conducted. Thirdly, this study employed a retrospective cohort design, which may have been subject to selection bias and recall bias. NVSS birth data are collected with the use of interpolation and bias control techniques in order to address the issue of missing information and are then entered and uploaded by staff who have undergone professional training. Prior to release to the public, each data item is subjected to internal consistency and edit checks, as well as a missing data check. This process serves to minimize the occurrence of bias to a certain extent. Fourthly, the NVSS birth data do not allow for the tracking of consecutive pregnancy records for the same mother to distinguish whether a previous caesarean section was the result of a previous or earlier pregnancy, and therefore mothers with a history of previous caesarean sections were excluded. This study employed several sensitivity analyses that were conducted to ascertain the stability and reliability of the results. Thus, as an observational study, it was not possible to ascertain a causal relationship between IPI and the risk of PTB in a population that had undergone vaginal delivery. Further validation is needed in other national populations, as this study population was all from the United States.

## Conclusion

In this large cohort study, a J-shaped association between IPI after vaginal delivery and singleton PTB was found, with ≤ 11 months and ≥ 24 months of IPI associated with an increased risk of PTB, but more studies are needed to further validate the analysis. Considering that IPI is a modifiable risk factor for PTB, it is recommended that reasonable guidance be given to patients during antenatal check-ups and the postnatal period.

## Supplementary Information

Supplementary Material 1.

## Data Availability

The datasets generated and analyzed during the current study are available in the NVSS database (https://www.cdc.gov/nchs/nvss/index.htm).

## Abbreviations

IPI: Interpregnancy interval
PTB: Preterm birth
RCS: Restricted cubic spline
WHO: World Health Organization
NVSS: National Vital Statistics System
NCHS: National Center for Health Statistics
CDC: Centers for Disease Control and Prevention
STROBE: Strengthening the Reporting of Observational Studies in Epidemiology
BMI: Body mass index
ART: Assisted reproductive technology
IOM: Institute of Medicine
DAG: Directed acyclic graph
OE: Obstetric estimates
BO-EDD: Best obstetric date of delivery
LMP: last menstrual period
ACOG: American College of Obstetricians and Gynecologists
SGA: Small for gestational age
LGA: Large for gestational age
SD: Standard deviation
ANOVA: One-way analysis of variance
OR: Odds ratio
CI: Confidence interval
Ref: Reference
