# Supplementary material for: Non-linear association between interpregnancy interval after vaginal delivery and singleton preterm birth: a retrospective cohort study
Source: BMC Pregnancy Childbirth. 2025 Mar 11;25:275. doi: 10.1186/s12884-025-07373-x (PMC11900573; doi:10.1186/s12884-025-07373-x)
Supplement: Supplementary file 1 — Supplementary Material 1. [file 12884_2025_7373_MOESM1_ESM.pdf]

Supplement Content

Supplementary Figure 1. Directed acyclic graph (DAG) illustrating confounder selection.

Supplementary Figure 2. Subgroup analyses between interpregnancy interval and preterm birth by age at the prior birth.

Supplementary Figure 3. Subgroup analyses between interpregnancy interval and preterm birth by pre-pregnancy BMI.

Supplementary Figure 4. Subgroup analyses between interpregnancy interval and preterm birth by parity before the current pregnancy.

Supplementary Figure 5. Subgroup analyses between interpregnancy interval and preterm birth by mode of delivery.

Supplementary Figure 6. Non-linear association between interpregnancy interval and preterm birth based on without potential confounders or missing data.

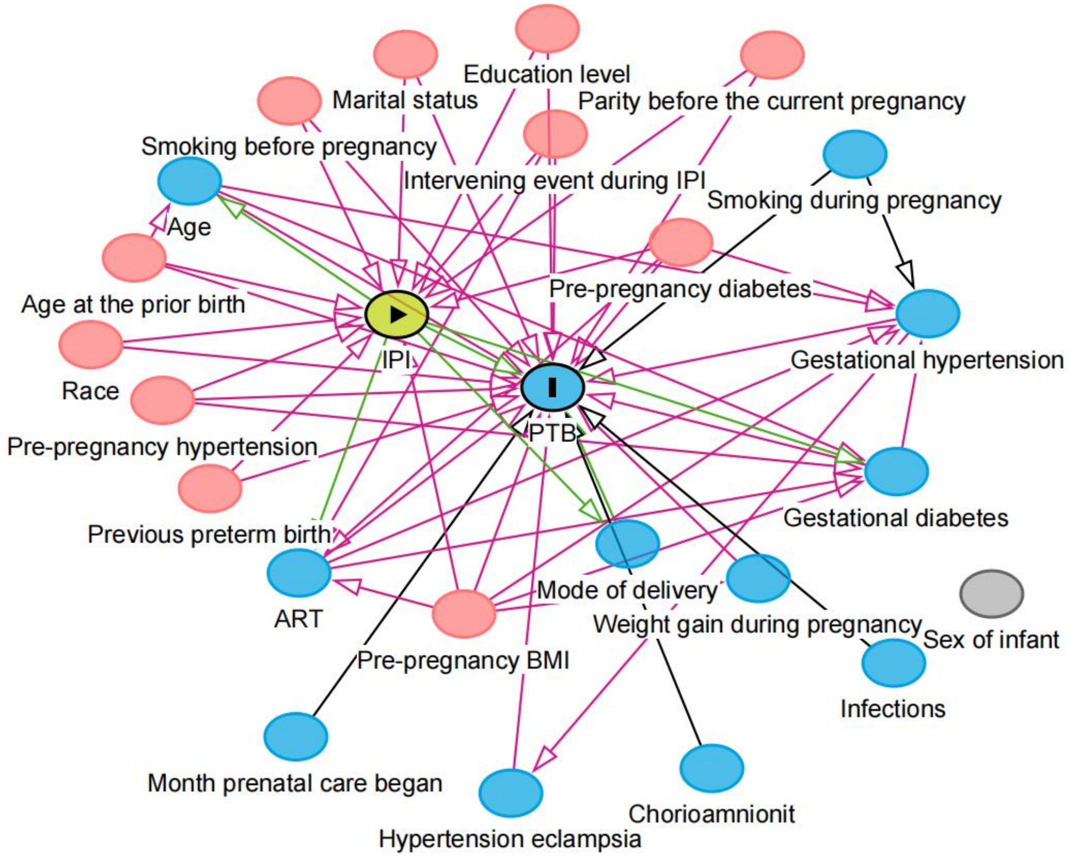

Supplementary Figure 1. Directed acyclic graph (DAG) illustrating confounders selection.

Abbreviations: IPI, interpregnancy interval; BMI, body mass index; ART, assisted reproductive technology.

Subgroup	n.Total	Moderate to late preterm birth n.Event%	Moderate to late preterm birth OR(95%CI)	Very preterm birth n.Event%	Very preterm birth OR(95%CI)	Extremely preterm birth n.Event%	Extremely preterm birth OR(95%CI)
**Age at the prior birth**	**1517106**						
**<35 years**	**1434747**						
<6 months	72394	6712 (9.3)	1.58 (1.53~1.63)	574 (0.8)	1.83 (1.63~2.05)	250 (0.3)	1.99 (1.67~2.38)
6–11 months	167779	10482 (6.2)	1.15 (1.11~1.18)	813 (0.5)	1.26 (1.13~1.40)	341 (0.2)	1.34 (1.14~1.58)
12–17 months	199153	10578 (5.3)	1.02 (0.99~1.05)	704 (0.4)	0.98 (0.88~1.10)	309 (0.2)	1.10 (0.93~1.31)
18–23 months	168883	8844 (5.2)	1(Ref)	610 (0.4)	1(Ref)	240 (0.1)	1(Ref)
24–35 months	235970	13757 (5.8)	1.09 (1.06~1.12)	977 (0.4)	1.11 (1.00~1.23)	420 (0.2)	1.20 (1.02~1.40)
36–59 months	265754	17328 (6.5)	1.17 (1.14~1.20)	1368 (0.5)	1.30 (1.18~1.43)	602 (0.2)	1.40 (1.21~1.63)
⩾60 months	324814	27954 (8.6)	1.50 (1.46~1.54)	2729 (0.8)	2.01 (1.84~2.20)	1252 (0.4)	2.20 (1.92~2.53)
**⩾35 years**	**82359**						
<6 months	4877	501 (10.3)	1.43 (1.27~1.62)	64 (1.3)	2.59 (1.77~3.78)	27 (0.6)	2.67 (1.48~4.80)
6–11 months	13980	956 (6.8)	1.08 (0.98~1.19)	63 (0.5)	1.08 (0.74~1.57)	38 (0.3)	1.60 (0.93~2.76)
12–17 months	17855	1151 (6.4)	1.09 (0.99~1.20)	90 (0.5)	1.32 (0.94~1.87)	42 (0.2)	1.55 (0.91~2.64)
18–23 months	13406	788 (5.9)	1(Ref)	51 (0.4)	1(Ref)	20 (0.1)	1(Ref)
24–35 months	16565	1187 (7.2)	1.24 (1.13~1.36)	97 (0.6)	1.55 (1.10~2.18)	44 (0.3)	1.79 (1.05~3.04)
36–59 months	11861	1080 (9.1)	1.52 (1.38~1.67)	79 (0.7)	1.63 (1.14~2.33)	34 (0.3)	1.80 (1.03~3.14)
⩾60 months	3815	408 (10.7)	1.73 (1.52~1.96)	34 (0.9)	2.05 (1.32~3.19)	15 (0.4)	2.41 (1.23~4.73)

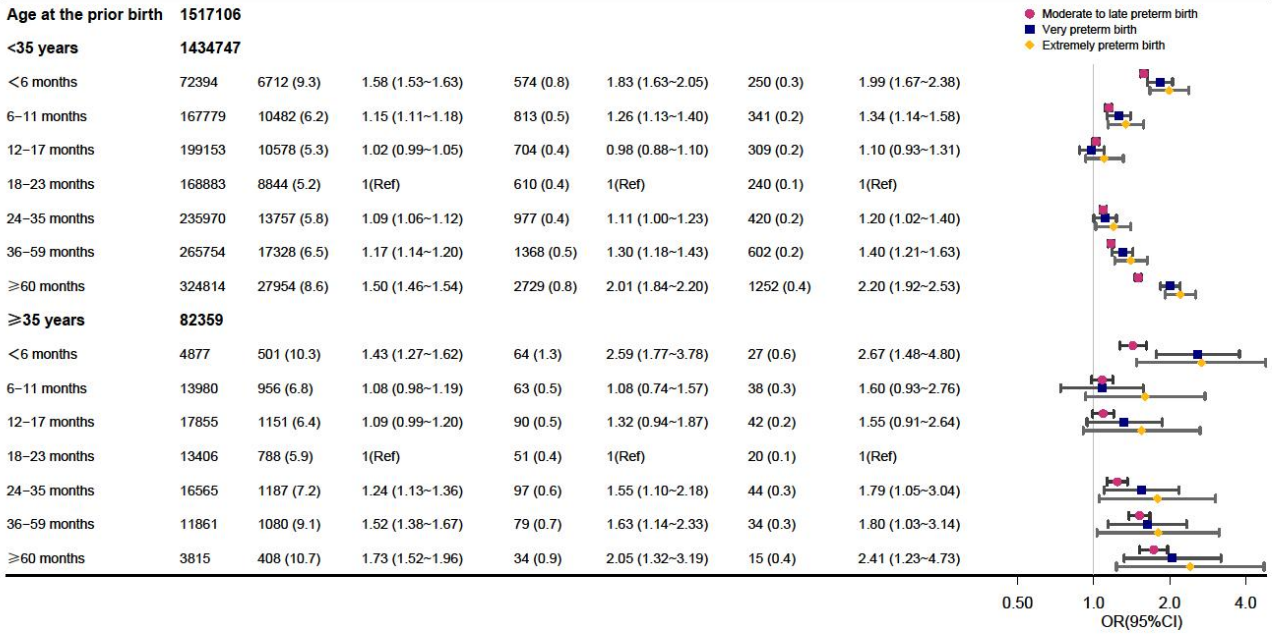

Supplementary Figure 2. Subgroup analyses between interpregnancy interval and preterm birth by age at the prior birth.

Abbreviations: CI, confidence interval; OR, odds ratio; Ref, reference; BMI, body mass index.

Adjusted for race, marital status, education level, intervening event during IPI, parity before the current pregnancy, pre-pregnancy BMI, smoking before pregnancy, pre-pregnancy diabetes, pre-pregnancy hypertension, and previous history of preterm birth.

Subgroup	n.Total	Moderate to late preterm birth n.Event%	Moderate to late preterm birth OR(95%CI)	Very preterm birth n.Event	Very preterm birth OR(95%CI)	Extremely preterm birth n.Event%	Extremely preterm birth OR(95%CI)
**BMI**	**1517106**						
**Underweight**	**37410**						
<6 months	1737	303 (17.4)	2.07 (1.75~2.45)	28 (1.6)	2.40 (1.41~4.07)	12 (0.7)	1.95 (0.91~4.17)
6–11 months	5021	525 (10.5)	1.33 (1.16~1.54)	63 (1.3)	2.08 (1.33~3.26)	26 (0.5)	1.61 (0.86~3.02)
12–17 months	6433	547 (8.5)	1.15 (1.00~1.32)	46 (0.7)	1.32 (0.83~2.11)	25 (0.4)	1.31 (0.70~2.46)
18–23 months	5140	387 (7.5)	1(Ref)	29 (0.6)	1(Ref)	16 (0.3)	1(Ref)
24–35 months	6743	589 (8.7)	1.13 (0.98~1.29)	49 (0.7)	1.25 (0.79~1.99)	24 (0.4)	1.09 (0.58~2.05)
36–59 months	6455	558 (8.6)	1.01 (0.88~1.16)	48 (0.7)	1.12 (0.70~1.78)	22 (0.3)	0.89 (0.46~1.70)
≥60 months	5881	582 (9.9)	1.11 (0.96~1.27)	64 (1.1)	1.51 (0.96~2.36)	24 (0.4)	0.96 (0.50~1.83)
**Normal weight**	**572996**						
<6 months	25281	2491 (9.9)	1.72 (1.63~1.82)	256 (1.0)	2.34 (1.96~2.79)	93 (0.4)	1.99 (1.50~2.64)
6–11 months	72727	4369 (6.0)	1.21 (1.15~1.26)	334 (0.5)	1.31 (1.11~1.55)	138 (0.2)	1.29 (1.00~1.67)
12–17 months	95424	4656 (4.9)	1.05 (1.00~1.10)	338 (0.4)	1.13 (0.96~1.33)	125 (0.1)	1.00 (0.77~1.30)
18–23 months	78834	3714 (4.7)	1(Ref)	252 (0.3)	1(Ref)	106 (0.1)	1(Ref)
24–35 months	102009	5414 (5.3)	1.09 (1.04~1.14)	404 (0.4)	1.18 (1.01~1.38)	164 (0.2)	1.13 (0.88~1.44)
36–59 months	99127	6061 (6.1)	1.16 (1.11~1.21)	474 (0.5)	1.28 (1.10~1.49)	181 (0.2)	1.14 (0.89~1.45)
≥60 months	99594	7923 (8.0)	1.42 (1.37~1.48)	740 (0.7)	1.82 (1.57~2.11)	310 (0.3)	1.79 (1.43~2.24)
**Overweight**	**432098**						
<6 months	22053	1888 (8.6)	1.54 (1.44~1.64)	144 (0.7)	1.59 (1.27~1.99)	72 (0.3)	2.24 (1.59~3.16)
6–11 months	50690	2850 (5.6)	1.07 (1.01~1.13)	239 (0.5)	1.26 (1.04~1.54)	100 (0.2)	1.50 (1.09~2.06)
12–17 months	58138	2871 (4.9)	0.97 (0.91~1.02)	194 (0.3)	0.93 (0.76~1.14)	77 (0.1)	1.05 (0.75~1.47)
18–23 months	49559	2509 (5.1)	1(Ref)	176 (0.4)	1(Ref)	62 (0.1)	1(Ref)
24–35 months	70751	3950 (5.6)	1.09 (1.03~1.15)	288 (0.4)	1.12 (0.92~1.35)	112 (0.2)	1.23 (0.90~1.68)
36–59 months	80429	4891 (6.1)	1.16 (1.10~1.22)	389 (0.5)	1.27 (1.06~1.52)	169 (0.2)	1.57 (1.17~2.10)
≥60 months	100478	8051 (8.0)	1.52 (1.45~1.60)	786 (0.8)	2.02 (1.71~2.39)	370 (0.4)	2.69 (2.04~3.55)
**Obesity**	**474602**						
<6 months	28200	2531 (9.0)	1.41 (1.33~1.49)	210 (0.7)	1.64 (1.35~1.99)	100 (0.4)	2.09 (1.55~2.83)
6–11 months	53321	3694 (6.9)	1.09 (1.04~1.15)	240 (0.5)	1.03 (0.85~1.24)	115 (0.2)	1.32 (0.99~1.77)
12–17 months	57013	3655 (6.4)	1.03 (0.98~1.08)	216 (0.4)	0.89 (0.74~1.08)	124 (0.2)	1.38 (1.04~1.84)
18–23 months	48756	3022 (6.2)	1(Ref)	204 (0.4)	1(Ref)	76 (0.2)	1(Ref)
24–35 months	73032	4991 (6.8)	1.11 (1.06~1.16)	333 (0.5)	1.09 (0.92~1.30)	164 (0.2)	1.43 (1.09~1.88)
36–59 months	91604	6898 (7.5)	1.23 (1.17~1.28)	536 (0.6)	1.41 (1.20~1.66)	264 (0.3)	1.82 (1.41~2.35)
≥60 months	122676	11806 (9.6)	1.62 (1.55~1.69)	1173 (1.0)	2.41 (2.07~2.81)	563 (0.5)	2.95 (2.31~3.77)

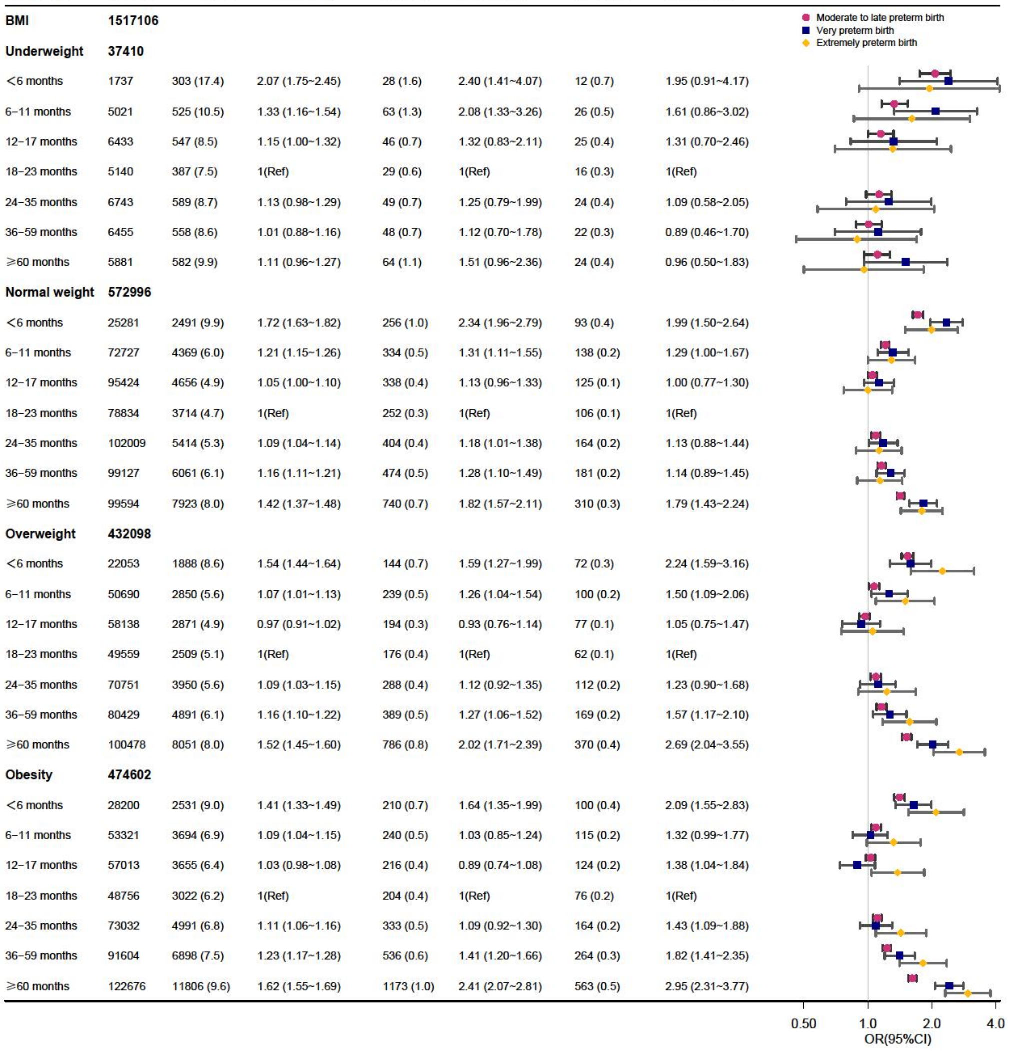

Supplementary Figure 3. Subgroup analyses between interpregnancy interval and preterm birth by pre-pregnancy BMI.

Abbreviations: CI, confidence interval; OR, odds ratio; Ref, reference; BMI, b ody mass index.

Adjusted for age at the prior birth, race, marital status, education level, intervening event during IPI, parity before the current pregnancy, smoking before pregnancy, pre-pregnancy diabetes, pre-pregnancy hypertension, and previous history of preterm birth.

Subgroup	n.Total	Moderate to late preterm birth n.Event%	Moderate to late preterm birth OR(95%CI)	Very preterm birth n.Event%	Very preterm birth OR(95%CI)	Extremely preterm birth n.Event%	Extremely preterm birth OR(95%CI)
**Parity before the current pregnancy**	**1517106**						
**1**	**789292**						
<6 months	35353	2684 (7.6)	1.52 (1.44~1.60)	244 (0.7)	2.10 (1.79~2.46)	123 (0.3)	2.32 (1.81~2.99)
6–11 months	93611	4913 (5.2)	1.11 (1.06~1.16)	370 (0.4)	1.16 (1.00~1.34)	168 (0.2)	1.12 (0.88~1.43)
12–17 months	118823	5526 (4.7)	1.02 (0.98~1.06)	361 (0.3)	0.89 (0.77~1.03)	170 (0.1)	1.10 (0.87~1.39)
18–23 months	100696	4677 (4.6)	1(Ref)	302 (0.3)	1(Ref)	126 (0.1)	1(Ref)
24–35 months	136265	7248 (5.3)	1.11 (1.07~1.15)	486 (0.4)	1.06 (0.92~1.22)	226 (0.2)	1.17 (0.94~1.47)
36–59 months	140276	8500 (6.1)	1.22 (1.17~1.26)	688 (0.5)	1.31 (1.15~1.49)	284 (0.2)	1.48 (1.20~1.83)
⩾60 months	164268	13150 (8)	1.55 (1.49~1.60)	1313 (0.8)	2.05 (1.81~2.32)	651 (0.4)	2.54 (2.09~3.10)
**2**	**406428**						
<6 months	19903	1847 (9.3)	1.63 (1.53~1.74)	147 (0.7)	1.73 (1.38~2.17)	66 (0.3)	2.32 (1.62~3.33)
6–11 months	44804	2812 (6.3)	1.16 (1.10~1.23)	215 (0.5)	1.21 (0.99~1.49)	94 (0.2)	1.61 (1.15~2.25)
12–17 months	51068	2826 (5.5)	1.05 (0.99~1.11)	197 (0.4)	1.01 (0.83~1.25)	81 (0.2)	1.28 (0.91~1.80)
18–23 months	44724	2345 (5.2)	1(Ref)	168 (0.4)	1(Ref)	55 (0.1)	1(Ref)
24–35 months	65903	3828 (5.8)	1.09 (1.03~1.15)	277 (0.4)	1.10 (0.91~1.33)	120 (0.2)	1.44 (1.04~1.98)
36–59 months	80090	5099 (6.4)	1.16 (1.10~1.22)	374 (0.5)	1.18 (0.98~1.42)	182 (0.2)	1.71 (1.26~2.32)
⩾60 months	99936	8643 (8.6)	1.53 (1.46~1.61)	813 (0.8)	1.99 (1.68~2.36)	359 (0.4)	2.58 (1.93~3.45)
**⩾3**	**321386**						
<6 months	22015	2682 (12.2)	1.60 (1.51~1.70)	247 (1.1)	1.89 (1.56~2.29)	88 (0.4)	1.64 (1.20~2.22)
6–11 months	43344	3713 (8.6)	1.17 (1.11~1.24)	291 (0.7)	1.22 (1.02~1.47)	117 (0.3)	1.20 (0.90~1.60)
12–17 months	47117	3377 (7.2)	1.02 (0.97~1.08)	236 (0.5)	0.98 (0.81~1.18)	100 (0.2)	1.01 (0.75~1.36)
18–23 months	36869	2610 (7.1)	1(Ref)	191 (0.5)	1(Ref)	79 (0.2)	1(Ref)
24–35 months	50367	3868 (7.7)	1.08 (1.02~1.14)	311 (0.6)	1.18 (0.99~1.42)	118 (0.2)	1.08 (0.81~1.43)
36–59 months	57249	4809 (8.4)	1.16 (1.10~1.22)	385 (0.7)	1.25 (1.05~1.49)	170 (0.3)	1.31 (1.00~1.72)
⩾ 60 months	64425	6569 (10.2)	1.45 (1.38~1.52)	637 (1)	1.92 (1.62~2.26)	257 (0.4)	1.83 (1.42~2.37)

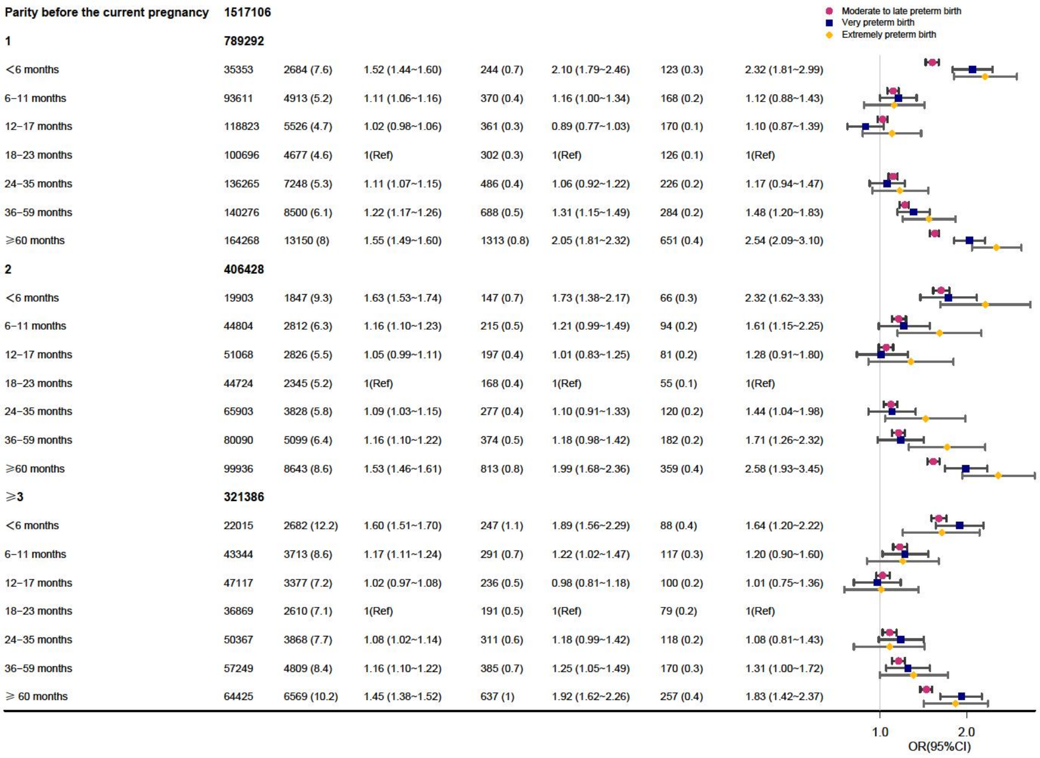

Supplementary Figure 4. Subgroup analyses between interpregnancy interval and preterm birth by parity before the current pregnancy.

Abbreviations: CI, confidence interval; OR, odds ratio; Ref, reference; BMI, body mass index.

Adjusted for age at the prior birth, race, marital status, education level, intervening event during IPI, pre-pregnancy BMI, smoking before pregnancy, pre-pregnancy diabetes, pre-pregnancy hypertension, and previous history of preterm birth.

Subgroup	n.Total	Moderate to late preterm birth n.Event%	Moderate to late preterm birth OR(95%CI)	Very preterm birth n.Event%	Very preterm birth OR(95%CI)	Extremely preterm birth n.Event%	Extremely preterm birth OR(95%CI)
**Mode of delivery**	**1517106**						
**Vaginal**	**1328095**						
<6 months	69603	5933 (8.5)	1.67 (1.61~1.73)	366 (0.5)	2.58 (2.2~3.03)	115 (0.2)	4.19 (3.02~5.81)
6−11 months	164537	9005 (5.5)	1.17 (1.13~1.21)	427 (0.3)	1.54 (1.33~1.80)	158 (0.1)	2.92 (2.14~3.99)
12−17 months	196395	9068 (4.6)	1.04 (1.01~1.07)	376 (0.2)	1.20 (1.03~1.40)	127 (0.1)	2.00 (1.45~2.76)
18−23 months	163821	7260 (4.4)	1(Ref)	286 (0.2)	1(Ref)	91 (0.1)	1(Ref)
24−35 months	224050	11165 (5.0)	1.09 (1.06~1.13)	434 (0.2)	1.15 (0.99~1.34)	163 (0.1)	2.10 (1.54~2.87)
36−59 months	241481	13211 (5.5)	1.15 (1.11~1.18)	595 (0.2)	1.39 (1.21~1.61)	200 (0.1)	2.17 (1.60~2.95)
⩾60 months	268208	18852 (7.0)	1.45 (1.41~1.49)	985 (0.4)	1.90 (1.65~2.18)	387 (0.1)	3.16 (2.36~4.23)
**C−Section**	**188267**						
<6 months	7624	1277 (16.7)	1.27 (1.18~1.37)	271 (3.6)	1.55 (1.32~1.82)	161 (2.1)	2.01 (1.61~2.50)
6−11 months	17121	2422 (14.1)	1.08 (1.01~1.15)	449 (2.6)	1.21 (1.05~1.39)	221 (1.3)	1.31 (1.07~1.60)
12−17 months	20468	2656 (13.0)	1.00 (0.94~1.06)	418 (2.0)	0.99 (0.86~1.14)	224 (1.1)	1.17 (0.96~1.43)
18−23 months	18369	2366 (12.9)	1(Ref)	374 (2.0)	1(Ref)	169 (0.9)	1(Ref)
24−35 months	28366	3768 (13.3)	1.02 (0.96~1.07)	639 (2.3)	1.07 (0.94~1.22)	301 (1.1)	1.10 (0.91~1.33)
36−59 months	36025	5188 (14.4)	1.09 (1.03~1.15)	850 (2.4)	1.09 (0.96~1.23)	435 (1.2)	1.20 (1.00~1.44)
⩾60 months	60294	9499 (15.8)	1.19 (1.14~1.26)	1776 (2.9)	1.32 (1.18~1.49)	878 (1.5)	1.39 (1.17~1.64)
**Missing**	**744**						

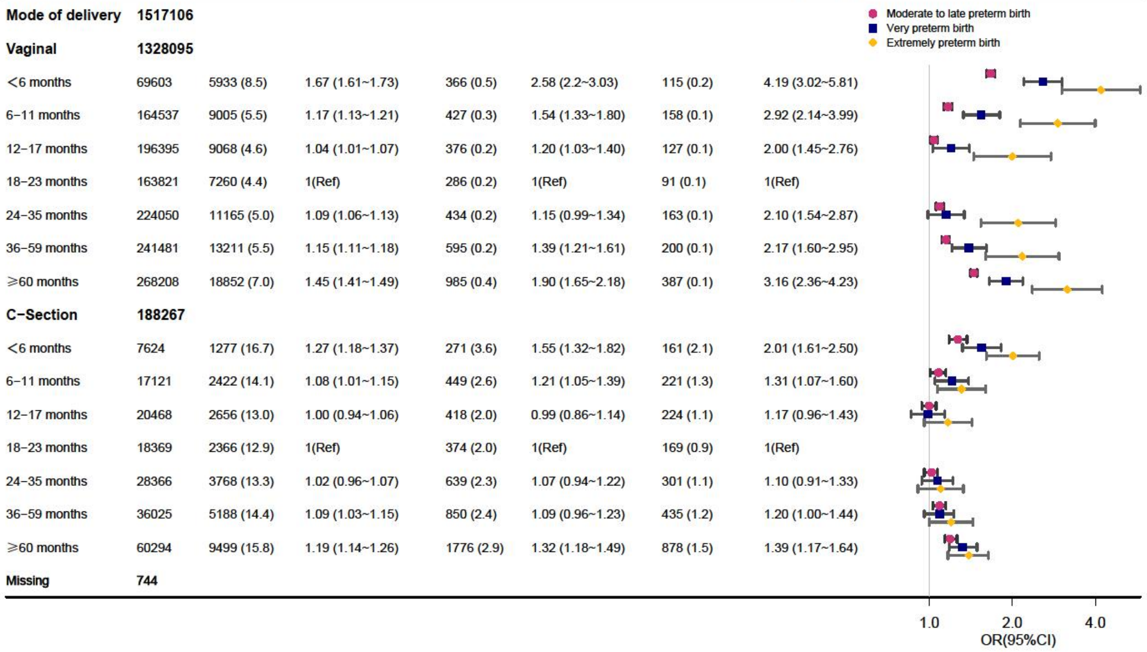

Supplementary Figure 5. Subgroup analyses between interpregnancy interval and preterm birth by mode of delivery.

Abbreviations: CI, confidence interval; OR, odds ratio; Ref, reference; BMI, body mass index.

Adjusted for age at the prior birth, race, marital status, education level, intervening event during IPI, parity before the current pregnancy, pre-pregnancy BMI, smoking before pregnancy, pre-pregnancy diabetes, pre-pregnancy hypertension, and previous history of preterm birth.

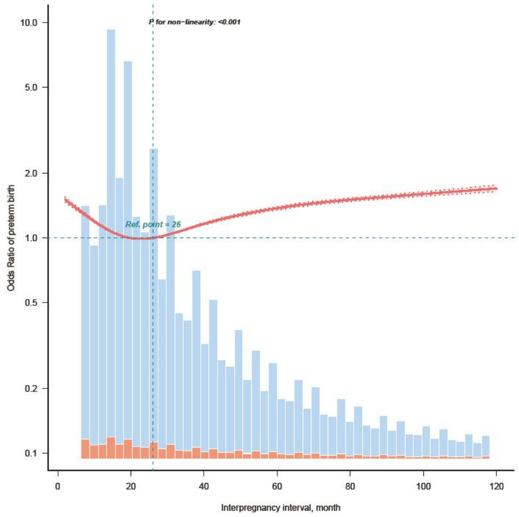
P for non−linearity: <0.001
Ref. point = 26
Odds Ratio of preterm birth
10.0
5.0
2.0
1.0
0.5
0.2
0.1
0
20
40
60
80
100
120
Interpregnancy interval, month

A

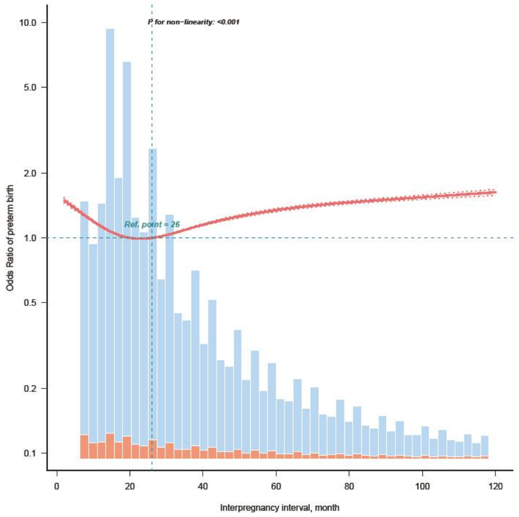
P for non−linearity: <0.001
Ref. point = 26
Odds Ratio of preterm birth
10.0
5.0
2.0
1.0
0.5
0.2
0.1
0
20
40
60
80
100
120
Interpregnancy interval, month

B

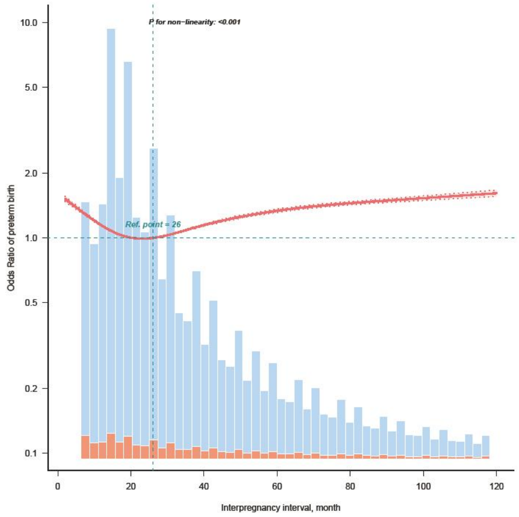
P for non−linearity: <0.001
Ref. point = 26
Odds Ratio of preterm birth
10.0
5.0
2.0
1.0
0.5
0.2
0.1
0
20
40
60
80
100
120
Interpregnancy interval, month

C

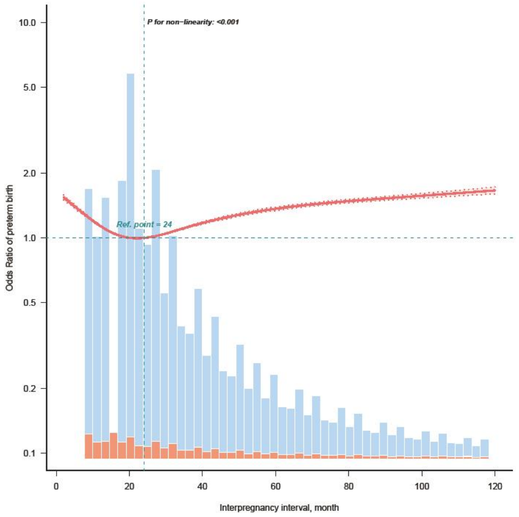
P for non−linearity: <0.001
Ref. point = 24
Odds Ratio of preterm birth
10.0
5.0
2.0
1.0
0.5
0.2
0.1
0
20
40
60
80
100
120
Interpregnancy interval, month

D

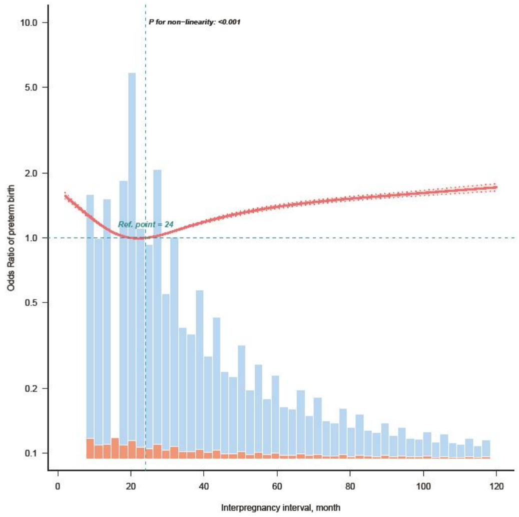
P for non−linearity: <0.001
Ref. point = 24
Odds Ratio of preterm birth
10.0
5.0
2.0
1.0
0.5
0.2
0.1
0
20
40
60
80
100
120
Interpregnancy interval, month

E

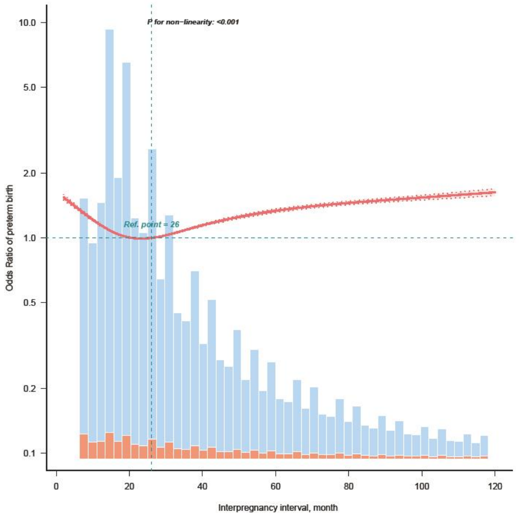
P for non−linearity: <0.001
Ref. point = 26
Odds Ratio of preterm birth
10.0
5.0
2.0
1.0
0.5
0.2
0.1
0
20
40
60
80
100
120
Interpregnancy interval, month

F

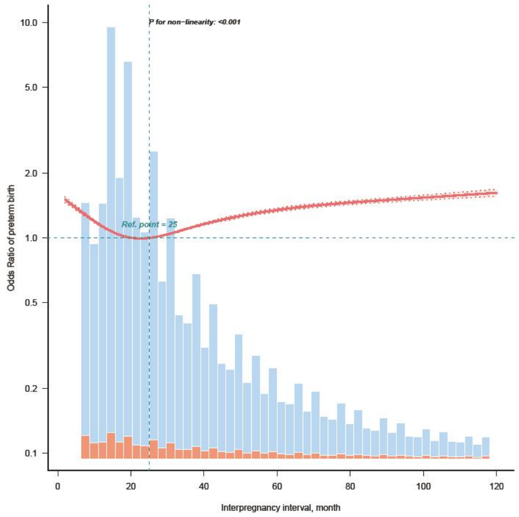

G

Supplementary Figure 6. Non-linear association between interpregnancy interval and preterm birth based on without potential confounders or missing data.

A: Without previous preterm birth (n=1364011)

B: Without pre-pregnancy diabetes (n=1426096)

C: Without pre-pregnancy hypertension (n=1403565)

D: Without intervening event during IPI (n=1262150)

E: Without previous preterm birth, pre-pregnancy diabetes, pre-pregnancy hypertension, and intervening event during IPI (n=1164858)

F: Without SGA or LGA (n=1155456)

G: Without missing covariates (n=1126842)

Abbreviations: CI, confidence interval; OR, odds ratio; Ref, reference; IPI, interpregnancy interval; BMI, body mass index; SGA, small for gestational age; LGA, large for gestational age.

Age at the prior birth, race, marital status, education level, intervening event during IPI, parity before the current pregnancy, pre-pregnancy BMI, smoking before pregnancy, pre-pregnancy diabetes, pre-pregnancy hypertension, and previous history of preterm birth were adjusted for in the model except when the variable was excluded.

Solid and dashed lines indicate the predicted value and 95% CI. Only data for the IPI in the range of 1-120 months are shown.
